# The diagnostic performance of automatic B-lines detection for evaluating pulmonary edema in the emergency department among novice point-of-care ultrasound practitioners

**DOI:** 10.1007/s10140-025-02319-4

**Published:** 2025-02-14

**Authors:** Kamonwon Ienghong, Lap Woon Cheung, Dhanu Gaysonsiri, Korakot Apiratwarakul

**Affiliations:** 1 Department of Emergency Medicine, Faculty of Medicine, Khon Kaen University, Khon Kaen, 40002 Thailand; 2 Accident & Emergency Department, Princess Margaret Hospital, Kowloon, Hong Kong China; 3 Department of Emergency Medicine, Li Ka Shing Faculty of Medicine, The University of Hong Kong, Pokfulam, Hong Kong China; 4 Department of Pharmacology, Faculty of Medicine, Khon Kaen University, Khon Kaen, Thailand

**Keywords:** Artificial intelligence, Emergency department, Ultrasound, Pulmonary edema

## Abstract

**Purpose:**

B-lines in lung ultrasound have been a critical clue for detecting pulmonary edema. However, distinguishing B-lines from other artifacts is a challenge, especially for novice point of care ultrasound (POCUS) practitioners. This study aimed to determine the efficacy of automatic detection of B-lines using artificial intelligence (Auto B-lines) for detecting pulmonary edema.

**Methods:**

A retrospective study was conducted on dyspnea patients treated at the emergency department between January 2023 and June 2024. Ultrasound documentation and electronic emergency department medical records were evaluated for sensitivity, specificity, positive likelihood ratio, and negative likelihood ratio of auto B-lines in detection of pulmonary edema.

**Results:**

Sixty-six patients with a final diagnosis of pulmonary edema were enrolled, with 54.68% having positive B-lines in lung ultrasound. Auto B-lines had 95.6% sensitivity (95% confidence interval [CI]: 0.92–0.98) and 77.2% specificity (95% CI: 0.74–0.80). Physicians demonstrated 82.7% sensitivity (95% CI: 0.79–0.97) and 63.09% sensitivity (95% CI: 0.58–0.69).

**Conclusion:**

The auto B-lines were highly sensitive in diagnosing pulmonary edema in novice POCUS practitioners. The clinical integration of physicians and artificial intelligence enhances diagnostic capabilities.

## Introduction

Acute pulmonary edema is a common and potentially fatal condition that often requires hospitalization in the emergency department [1–3]. Symptoms of pulmonary edema may develop suddenly or gradually, varying with the type of pulmonary edema. Clinical characteristics can be atypical [4, 5]. Assessing pulmonary edema has remained a diagnostic challenge particularly for novice point of care ultrasound (POCUS) practitioners [6, 7].

B-lines are discrete short path vertical reverberation artifacts that manifest as echogenic lines extending from the pleural line into the lung parenchyma without diminishing in intensity and move in synchrony with lung sliding. An elevation in the density of the underlying lung due to the substitution of air with exudate, transudate, collagen, blood, etc., diminishes the acoustic mismatch between the pleura and the underlying structures. This results in the reflection of the ultrasound beam back to the transducer, creating a bidirectional movement of the reflected beam, which generates characteristic comet-tail artifacts [8]. B-lines correlate with extravascular lung water content in addition to conventional methods such as medical examinations, biomarkers, and chest radiographs in evaluating extravascular lung fluid in acute settings, assisting in the diagnosis of pulmonary edema [9–11]. Depending on the training, expertise in interpreting ultrasound, and clinical context, assessing B-lines can be challenging to accomplish and reproduce [11, 12].

The introduction of artificial intelligence (AI) in healthcare represents a turning point in medical innovation, extensively impacting various medical fields involved, including emergency medicine [13–19]. AI technology, such as Smart B-lines or Auto B-lines (automatic detection of B-lines using artificial intelligence) can detect and quantify lung B-lines, potentially reducing the need for operator intervention in ultrasound assessments and expanding lung ultrasound to novice operators [20, 21]. Previous study [21] demonstrated that The AI software (Auto B-Lines; GE Healthcare) exhibited a sensitivity of 95.6% (95% confidence interval [CI]: 0.92–0.97) and a specificity of 64.1% (95% CI: 0.59–0.68) in evaluating B-lines in pulmonary edema. Prior studies [22–24] revealed AI deep learning algorithm (Auto B-Lines), which quantified B-lines could detect fluid overload in dialysis patients. Moreover, auto B-lines assisted clinical scoring system to identify pulmonary congestion.

Following the difficulty of diagnosing pulmonary edema and the competency of B-lines interpretation, we hypothesized that auto B-lines, a revolutionary technology in ultrasound machine, could facilitate Thailand’s emergency medicine professionals’ diagnosis pulmonary edema. However, research in this area is still limited. In this study, we aimed to investigate the diagnostic performance of auto B-lines conducted by emergency medicine residents in identifying pulmonary edema in the emergency department.

## Methods

### Study design and population

This is a single center, retrospective study, a computerized medical record review was conducted from January 2023 to June 2024 at the Department of Emergency Medicine, Faculty of Medicine, Khon Kaen University. This hospital was the largest urban academic medical center in northeastern Thailand, serving 60,000 to 70,000 emergency patients annually. The primary purpose of this study was to investigate the diagnostic performance of auto B-lines in assisting emergency medicine residents in the diagnosis of pulmonary edema in emergency patients with dyspnea.

Patients admitted to the emergency department with dyspnea who received lung ultrasound examinations were recruited for this study. Eligibility criteria were based on all patients admitted to the emergency department with a final diagnosis of pulmonary edema as defined by the ICD-10 International Classification of Diseases, Code J81.0. Patients who had interstitial lung disease, lung tumor, and missing ultrasound documentation were disqualified from the study.

The sample size for the diagnostic test was determined based on an estimated prevalence of 0.36 with a standard normal value of 1.96 [25]. The power analysis was conducted with an alpha of 0.05. Sensitivity was 0.956, 1– sensitivity was 0.064, with an absolute precision of 0.01 [21]. This produced an estimated desired effect sample size of 66 subjects.

This study followed the principles of the Helsinki Declaration’s and the recommendations of Good Clinical Practice. The Ethics Committee for Human Research at Khon Kaen University authorized the study (HE671411). Approval from the institutional ethics committee was secured. The requirement for written informed consent from the patients was waived. The authors state that this report is devoid of any personal information that could facilitate the identification of the patients. The authors affirm that the patients’ personal information has been omitted.

### Data collection

Data collected and extracted from electronic medical records which included individual demographic data (age, sex, triage level, and past medical history), ultrasound characteristics of the patients including whether Auto B-lines were utilized in each case, and the final diagnosis of pulmonary edema according to ICD-10, Code J81.0.

This study classified emergency medicine residents as novice POCUS practitioners owing to their inexperience. They completed a one-month ultrasound rotation focused on comprehensive lung ultrasound. This course was a prerequisite for the residency training program.

Each patient was assessed by an individual emergency medicine resident, who acted as the treating physician in the emergency department at that moment. POCUS was routinely performed in patients suspected of pulmonary edema, encompassing ultrasound characteristics of the heart, lungs, and inferior vena cava. However, the decision to conduct POCUS at that time depended upon individuals. The ultrasound characteristics were documented in the medical record form.

This study routinely employed lung ultrasound utilizing the BLUE protocol [8], which is designed for diagnosing the etiology of acute respiratory failure. The patients were positioned supine. The scans were conducted longitudinally along three standardized points: the upper BLUE point, the lower BLUE point, and the PLAPS point. A-lines, B-lines, lung sliding, lung point, and lung consolidation were assessed and analyzed. Furthermore, venous analysis was conducted when the etiology of pulmonary embolism was suspected. According to the B-profile adhering to the BLUE protocol, the presence of three or more B-lines in a single view was classified as abnormal.

**Fig. 1:**
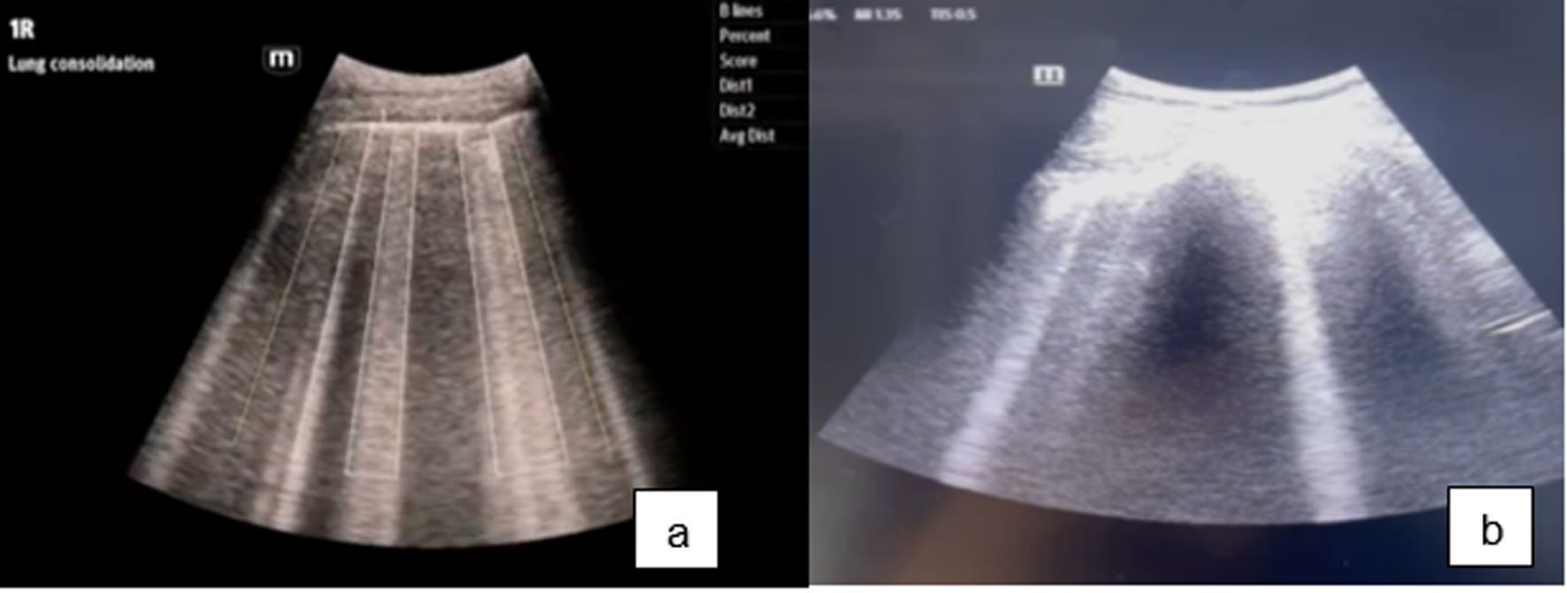
The performance of artificial intelligence (**a**) versus physician assessment (**b**) in detecting B-lines

Two methods were used to assess B-lines artifacts in this study. A method was selected based on the treating physician’s preference to be utilized concurrently with the physician performing the ultrasound. First, B-lines were enumerated through eye examination by the physician, who independently interpreted the findings. Second, B-lines were determined using the ultrasound machine’s automatic B-line function. This function, named “Smart B-lines” or “auto B line”, is an AI operation that automatically quantifies and analyzes B-lines (Fig. 1). The utilization of Auto B-lines in each patient was documented in the electronic medical record.

The ultrasound device employed in this research was the Mindray M9 (Mindray, Shenzhen, China), which was the main ultrasound machine employed in the resuscitation area of our department. The ultrasound probes employed for the identification of B-lines were the Mindray C5-1s curved array transducer, which operates within a frequency range of 1.0 MHz to 5.0 MHz. The configuration was designated for lung examination. Ultrasound video recordings were saved for a minimum of 6 s per clip in accordance with our hospital’s standards. The video and ultrasound images were stored in the machine, from which the investigators extracted the data using a Universal Serial Bus or USB drive.

The final diagnosis of pulmonary edema was defined by the ICD-10 International Classification of Diseases, 10th edition, Code J81.0. The ICD-10 was documented by the treating physician responsible for the case, who utilized clinical criteria to diagnose acute pulmonary edema, including jugular venous distension, pulmonary rales or crackles upon auscultation, and bilateral lower extremity edema. Furthermore, indicators of pulmonary edema on a standard chest radiograph were observed, including upper lobe pulmonary venous diversion, an elevated cardiothoracic ratio, Kerley lines, or pleural effusion, in conjunction with ground-glass opacification or interlobar septal thickening identified in computed tomography scans.

Data was collected and organized into a research database. Two independent investigators then reviewed the data, addressing any redundant entries. Null values were identified as missing values in the research data set. If columns had more than half of the rows as null, then the entire column can be dropped. If there were inconsistencies of the data, the senior investigator with more than ten years of experience in this field was consulted to obtain the correct data.

### Statistical analysis

Continuous variables in the data were summarized using mean and standard deviation or median and range, as appropriate. Categorical variables were summarized using counts and percentages. Diagnostic performance of auto B-lines and manual B-lines counted by physician visual inspection were determined as sensitivity, specificity, positive likelihood ratio, and negative likelihood ratio. The data were entered into Microsoft Excel and analyzed using IBM SPSS for Windows version 27.0, licensed to Khon Kaen University (SPSS Inc., Chicago, Illinois, USA).

## Results

### Baseline characteristics of studied cases

During the 18-month study period, 210 patients with dyspnea were admitted to the emergency department. One hundred forty-four patients were excluded. 42 had interstitial lung disease, 22 had lung tumors, and 80 were missing ultrasound documentation. A total of 66 patients with final diagnosis of pulmonary edema were enrolled in this study, with a mean age of 63.28 years, 51.51% of whom were women. The patient characteristics are shown in Table 1.

**Table 1:** Patient demographic information

Variables	Patient with suspected pulmonary edema (*N* = 66)
Age (year), median (IQR)	63.28 (51.13, 75.08)
Women, %	34 (51.51)
Triage level, %	
Resuscitation (1 and 2)	52 (78.78)
Urgent (3)	14 (21.21)
Past medical history, %	
Hypertension	45 (68.18)
Coronary artery disease	40 (60.60)
Asthma/ COPD	11 (16.66)
Kidney disease	21 (31.81)
Cirrhosis	6 (9.09)
Final diagnosis, %	
Pulmonary edema	35 (54.68)
Pneumonia	17 (25.75)
Asthma/ COPD exacerbation	13 (19.69)
Pulmonary embolism	1 (1.51)

Data are presented as median and Interquartile range (IQR) or frequency (%). SD: Standard deviation; COPD: Chronic Obstructive Pulmonary Disease

### Outcomes

Overall, 54.68% (*n* = 35) of the patients had B-lines in lung ultrasound. The use of automatic B-lines to assist physicians in diagnosing pulmonary edema (Table 2) demonstrated a sensitivity of 95.6% (95% CI: 0.92–0.98) and 77.2% specificity (95% CI: 0.74–0.80). The physician employed manual B-lines assessment, which exhibited 82.7% sensitivity (95% CI: 0.79–0.97) and 63.09% specificity (95% CI: 0.58–0.69).

**Table 2:** The diagnostic capability of automatic B-lines and physician assessment of pulmonary edema

Categorized	Sensitivity (95% CI)	Specificity (95% CI)	LR + (95% CI)	LR- (95% CI)
Automatic B-lines	95.6% (92.8–98.5)	77.2% (74.1–80.6)	4.19 (3.45–4.68)	0.06 (0.03–0.08)
Physician	82.7% (79.5–97.3)	63.09% (58.9–69.5)	2.24 (2.01–2.86)	0.27 (0.08–0.39)

Data are presented with 95% confidence interval. LR: likelihood ratio

## Discussion

There are many artifacts in lung ultrasound; nevertheless, B-lines represent a specific ultrasound artifact characterized by the following: they start at the pleural line, have a light ray-like appearance that obscures other background lung ultrasound artifacts, and move with lung sliding. These characteristics, however, make it difficult for inexperienced sonographers to distinguish B-lines from other vertical ultrasound artifacts, potentially leading to misdiagnosis of pathologic interstitial syndrome [26–28].

Nowadays, AI has developed specific automated capabilities to assist novice learners in quantifying B-lines. However, current applications have demonstrated moderate to fair agreement with experts at the binary or ordinal scoring levels [29, 30]. There remains little evidence to support the utility of combining automatic B-lines with physician judgement for detecting pulmonary edema.

Our study employed the lung ultrasound machine’s automatic B-lines function to assist the treating physician in the emergency department in distinguishing B-lines from other lung ultrasound artifacts and counting the number of B-lines. The use of automatic B-lines on lung ultrasound assisted the physician in diagnosing pulmonary edema with a sensitivity of 95.6% and a specificity of 77.2%. These findings are consistent with previous studies [21, 24, 30, 31] which established that B-lines are highly sensitive but nonspecific for evaluating lung disease because they are influenced by the distribution of B-lines and the patient’s clinical context, both of which must be interpreted by the attending physician’s clinical integration. However, one study in an emergency department with a population size similar to ours demonstrated that AI undercounted B-lines compared to expert assessment [32]. Another study involving a smaller population demonstrated the same outcome [33].

Our study additionally demonstrated that physicians who visualized B-lines and interpreted ultrasound findings independently had lower sensitivity and specificity than those who used automatic B-lines. This variation could be explained by the differing levels of ultrasound experience among our physicians. In contrast to our findings, a previous study showed no difference in diagnostic performance between senior radiologists, junior radiologists, and radiologists using AI [34]. However, that study focused on the use of AI to distinguish between benign and malignant thyroid nodules, which is distinct from our research.

Our study affirms the potential of AI for widespread application. Moreover, automatic B-lines are highly valuable in clinical practice, highlighting the capacity of AI to offer accurate decision support. AI might assist less experienced sonographers in enhancing their diagnostic abilities and minimizing misdiagnosis. This highlighted the fact that AI can provide cognitive assistance; however, physicians must interpret results within the clinical context of individual patients [35].

The study’s limitations were: (1) it was a single-center study, which may have introduced bias regarding the study population compared to other organizations, (2) this was a retrospective observational study, there may be biases related to patient selection which automatic B-lines were employed based on the physician’s preferences, and incomplete or imprecision data, (3) The knowledge and competence of the ultrasonographic operators were not assessed in this study; however, the POCUS practitioners underwent one month of ultrasound training. (4) The study used a specific ultrasound machine with automatic B-line functionality, so the findings may not be applicable to other ultrasound machines with varying capabilities, (5) our study used the final diagnosis as pulmonary edema following ICD-10 as the reference test which may affect the magnitude and direction of the effect of verification bias on the results, and (6) B-lines in the lung may signify conditions beyond pulmonary edema; however, our study did not encompass patients for whom automatic B-lines were employed in lung ultrasound for different diagnoses.

In conclusion, our findings demonstrate that automatic B-lines performed in lung ultrasound by emergency medicine residents exhibit high sensitivity for determining pulmonary edema. The information acquired through this AI might guide management decisions when treating emergency patients with dyspnea. Further research is required to externally validate and clarify the relationship between automated B-line measurements and other lung pathologies.

## Data Availability

The datasets used and/or analyzed during the current study are available from the corresponding author upon reasonable request.
